# Predictive value of serum transthyretin for outcome in acute ischemic stroke

**DOI:** 10.1371/journal.pone.0179806

**Published:** 2017-06-21

**Authors:** Wojciech Ambrosius, Slawomir Michalak, Radosław Kazmierski, Natalia Andrzejewska, Wojciech Kozubski

**Affiliations:** 1 Department of Neurology, Poznan University of Medical Sciences, Poznan, Poland; 2 Department of Neurochemistry and Neuropathology, Poznan University of Medical Sciences, Poznan, Poland; 3 Department of Neurology and Cerebrovascular Disorders, Ludwik Bierkowski Hospital, Poznan University of Medical Sciences, Poznan, Poland; University of Florida, UNITED STATES

## Abstract

**Introduction:**

The impact of choroid plexus with its blood–cerebrospinal fluid barrier in the ischemic stroke pathology is poorly explored. Transthyretin (TTR) is a protein synthesized in liver and just in choroid plexus.

**Objectives:**

The current study was designed to assess the prognostic value of serum TTR for functional outcome (at the time of hospital discharge) and long-term (one-year) overall mortality in ischemic stroke patients.

**Patients and methods:**

We conducted a prospective observational study. Patients (n = 81) with acute (< 24 hours of symptoms onset) ischemic stroke consecutively admitted to Stroke Unit were included. An unfavorable outcome was defined as a modified Rankin Scale (mRS) score ≥ 3. The relationships between serum TTR levels and clinical outcome were analyzed using multivariate analysis. One-year mortality was analyzed by Kaplan–Meier survival curves stratified by mean value of TTR.

**Results:**

Compared with patients with mRS <3, patients with an unfavorable outcome at hospital discharge had significantly lower TTR levels on admission (*P* < 0.0001). In non-survivals serum TTR levels were significantly lower compared with patients who survive one year of observation (*P =* 0.009). Using multivariate analysis, transthyretin emerged as an independent predictor for unfavorable outcome at the day of hospital discharge (adjusted odds ratio = 0.96; 95% CI: 0.9–0.99, *P* <0.05). A one-year mortality of patients with the lower TTR levels was significantly higher than in patients with TTR levels above mean value (*P* = 0.02).

**Conclusions:**

Serum level of TTR at admission was a predictor of functional outcome after ischemic stroke and was also associated with one-year mortality in stroke survivals.

## Introduction

Both, blood–brain barrier (BBB) and blood–cerebrospinal fluid barrier (BCSFB) maintain appropriate, stable, and well controlled environment which is essential for the central nervous system (CNS) functions. In the course of brain ischemia these interfaces may be damaged. Mechanisms of blood–brain barrier disruption in acute stroke are intensively studied for many years. Basic pathological phenomena are well explored: the increase in BBB permeability which precipitate extravasation of high molecular weight molecules, leukocyte infiltration (with e.g. MMP-9 release [1]), accumulation of various active molecules such as vascular endothelial growth factors [2], or bradykinin [3] and rearrangement of BBB tight-junctions proteins [4].

Surprisingly the probable link between brain ischemia and capabilities of BCSFB located in choroid plexus paid very little attention [5]. In humans together with the liver, choroid plexus synthetizes transthyretin (TTR) [6]. This protein is primarily known as a carrier of thyroxine (T4) and vitamin A (retinol). It plays a role in T4 transfer from cerebrospinal fluid (CSF) to the brain [7]. Some also had speculated that TTR might be important in transport of T4 from the bloodstream into CSF [8,9].

Alterations of the thyroid hormones metabolism are significant aspect of acute critical diseases as severe trauma, myocardial infarction, diabetic acidosis, thermal injury, sepsis, and stroke. Most of studies have focused on the role of free triiodothyronine (T3) in those conditions. It has been shown that low levels of this hormone (low fT3 syndrome) are predictor of poor outcome also in ischemic stroke [10,11]. Recently the association between thyroid stimulating hormone and stroke prognosis has been reported [12].

As TTR is involved in thyroid hormone transport we intended to explore the possible association between levels of this protein and outcome in ischemic stroke.

## Material and methods

### Study design

The study was approved by the Bioethics Committee of our university (decision no. 559/14). This was not intervention trial, nevertheless an informed consent was obtained from all subjects after sufficient and full explanation of the study objective (some patients were not able to give a consent, then it was taken from his close family i.e. spouse, relative in the ascending or in the descending line). All personal data of the analyzed patients were treated confidentially. Patients’ names and surnames were not included in the research records. Recruitment for this prospective ischemic stroke cohort study took place at the department of neurology in the university hospital. For inclusion, patients had to be admitted within 24 hours of experiencing a new focal or global neurological event. Acute ischemic stroke was defined according to the World Health Organization criteria [13]. Brain imaging (CT or MRI) was performed routinely within one hour after admission. Patients with an undetermined time of symptoms onset, malignant tumor, intracerebral hemorrhage, a history of recent surgery or trauma during the preceding three months, renal or liver failure, recent myocardial infarction, autoimmune or inflammatory diseases, known thyroid glands disease, were not included in the study.

Baseline characteristics with following variables were recorded: age, gender, waist-hip ratio, history of conventional vascular risk factors, as hypertension, previous stroke history, coronary artery disease, diabetes mellitus, atrial fibrillation, hyperlipoproteinemia, smoking habit and alcohol abuse. Routine blood and biochemical tests were performed in all patients at admission. Stroke severity was quantified using the National Institutes of Health Stroke Scale (NIHSS) at the admission and 7^th^ day of hospitalization. Acute ischemic stroke was classified according to the TOAST system, that differentiates large artery arteriosclerosis, small artery occlusion, cardioembolism, other demonstrated cause, and cryptogenic stroke [14].

The primary end point was a functional outcome assessment at the day of hospital discharge. The functional status was evaluated with the modified Rankin Scale (mRS) and the patients were classified into two groups: poor (mRS score of 3–6) and good (mRS score from 0 to 2) outcome. The secondary end point of this study was death from any cause within a 1-year follow- up. This outcome was achieved by a telephone interview with the patient or his/her relatives. Outcomes has been obtained by neurologists, blinded to clinical history and biomarker concentrations.

Blood and biochemical tests were taken on admission to hospital, within first 24 hours after stroke symptoms onset. Serum transthyretin levels were quantified by commercially available enzyme-linked immunosorbent assay (Abcam, Cambridge, UK) according to the manufacturer’s instructions. Samples were processed by the same laboratory technician with the same equipment and blinded to all clinical data.

### Statistical analysis

The results were reported as counts (percentage) for the categorical variables, mean±standard deviation if normally distributed, and median (interquartile range) if not normally distributed for the continuous variables.

Continuous variables were compared using independent sample t-tests and Mann–Whitney tests, and categorical variables using Chi-square tests or Fisher’s exact tests, as appropriate. Correlations among continuous variables were assessed by the Spearman rank-correlation coefficient. One-year overall survival was estimated using the Kaplan–Meier method by the log-rank test. The relations of TTR levels with the unfavorable outcome (assessed by mRS score) was investigated with the use of logistic regression models. We used univariate and multivariate models adjusted for all significant outcome predictors and report odds ratios (ORs) and 95% confidence interval (CI). Significance was set at P <0.05. All statistical analyses were performed using licensed MedCalc software for Windows (version 16.8.4).

## Results

Finally, 81 patients fulfilled inclusion and exclusion criteria and were enrolled to the study. The baseline characteristics of this population are described in Table 1. Overall mean age was 67 years, and 60% were men. An unfavorable functional outcome with mRS score ≥ 3 was found in 38 patients (47%). Through one year of observation 11 patients died, thus the mortality rate was 13.5%.

**Table 1:** Baseline characteristics of stroke patients.

Characteristic	All	Good outcome (mRS = 0–2)	Poor outcome (mRS = 3–6)	*P*
***n* (%)**	81	43 (53%)	38 (47%)	NS
**Female sex (*n*)**	32	15	17	NS
**Mean age (years)**	67 ± 14	63 ± 14	71 ± 14	<0.05
**Admission NIHSS score**	7 (4–14)	4(2–6)	13 (8–19)	<0.001
**Atrial fibrillation, *n* (%)**	28 (34%)	11 (25.5%)	17 (44.7%)	NS
**Hypertension, *n* (%)**	60 (74%)	29 (67.4%)	31 (81.5%)	NS
**Diabetes, *n* (%)**	22 (27%)	9 (20.9%)	13 (38.4%)	NS
**History of stroke, *n* (%)**	25 (30%)	13 (30.2%)	12 (31.5%)	NS
**Coronary heart disease, *n* (%)**	36 (44.8%)	18 (41.8%)	18 (47.3%)	NS
**Current cigarette smoking, *n* (%)**	18 (22%)	12 (27.9%)	6 (15.7%)	NS
**Stroke-associated infection**	15 (18.5%)	6 (13,9%)	9 (23,6%)	NS
**TOAST classification**				
** a. Large artery, *n* (%)**	24 (29%)	10 (23.2%)	14 (36.8%)	NS
** b. Small artery, *n* (%)**	11 (13.5%)	8 (18.6%)	3 (7.8%)	NS
** c. Cardioembolism, *n* (%)**	26 (32%)	10 (23.2%)	16 (42.1%)	NS
** d. Other cause, *n* (%)**	2 (2.5%)	1 (2.3%)	1 (2.6%)	NS
** e. Unknown, *n* (%)**	17 (20%)	13(30.2%)	4 (10.5%)	NS
**Glucose (mmol/L)**	5.5 (4.9–7.6)	5.2 (4.7–6.5)	5.8 (5.1–7.9)	<0.05
**Cholesterol (mg/dL)**	200 ± 52.2	209 ± 49.8	190.1 ± 53.7	NS
**HDL cholesterol, (mg/dL)**	50.2 ± 17.2	53.9 ± 16.8	46.2 ± 16.9	<0.05
**LDL cholesterol, (mg/dL)**	121.1 ± 45.3	123 ± 45.3	118 ± 47.7	NS
**Triglyceride, (mg/dl)**	126.8 ± 67.5	141.9 ± 79.6	110 ± 46.3	<0.05
**White cell count, ×109/L**	9.14 ± 3.3	8.3 ± 1.9	10 ± 4.2	<0.05
**Fibrinogen (mg/dL)**	401.4 ± 126	394.6 ± 135.6	408.4 ± 117.2	NS
**CRP (mg/l)**	3.3 (1.3–11.5)	2.3 (1.2–6.1)	7.2 (2.0–3.1)	<0.05
**fT3 (pg/ml)**	4.2 (3.27–4.82)	4.5 (4.1–4.92)	3.6 (2.45–4.2)	<0.001
**Transthyretin (mg/dL)**	46.3 (34.9–60.47)	53.3 (47.84–60.61)	37.32 (30.03–43.68)	<0.0001

All data are presented as median and interquartile ranges (IQR) or number (percentage).

CRP: C-reactive protein; fT3: free triiodothyronine; HDL: high density lipoprotein; LDL: low density lipoprotein; mRS: modified Rankin Scale; NIHSS: National Institute of Health Stroke Scale.

Lower TTR levels were significantly associated with the higher NIHSS score at admission–there was a negative correlation between levels of TTR and NIHSS (r = -0.41, *P* < 0.001). Analogously, TTR concentration negatively correlated with CRP levels (r = -0.31, *P* <0.01). Furthermore, TTR levels were higher with increasing total cholesterol and triglyceride levels (r = 0.33, P <0.01 and r = 0.24, P = 0.03, respectively).

**Fig 1:**
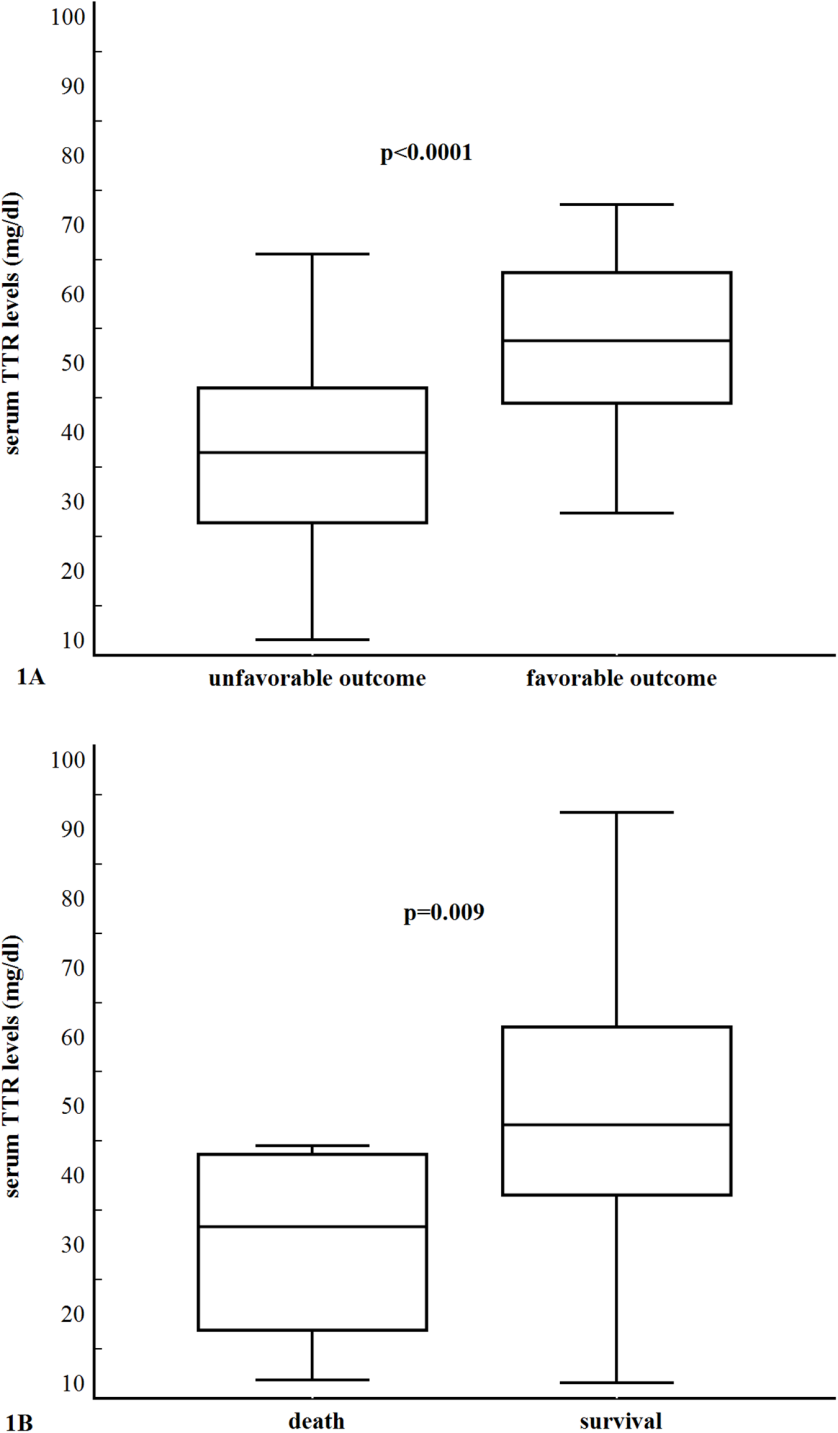
**(A). Serum transthyretin levels in stroke patients with favorable (mRS score <3) and unfavorable (mRS score** ≥ **3–6) outcomes. (B). Serum transthyretin levels in survivor and non-survival stroke patients.** All data are medians and interquartile ranges (IQR) with minimum and maximum values.

In the 38 patients with an unfavorable outcome, serum TTR levels were lower compared with those in 43 patients with a favorable functional outcome: 37.2 (IQR, 30.03–43.68) ng/ml vs. 53.3 (IQR, 47.84–60.61) ng/ml; *P* < 0.0001) (Fig 1A).

In univariate logistic regression we analyzed TTR, and other risk factors as presented in Table 2. With an unadjusted OR of 0.94 (95% CI, 0.9–0.97), TTR had a statistically significant (*P* = 0.0001) association with unfavorable functional outcome. After adjusting for all other significant outcome predictors (age, the NIHSS score, atrial fibrillation, small-vessel occlusive etiology, white cell count, and HDL cholesterol, triglyceride, CRP, fT3 levels) transthyretin was still an independent (*P* <0.05) outcome predictor with an adjusted OR of 0.96 (95% CI, 0.9–0.99).

**Table 2:** Univariate and multivariate logistic regression analysis for unfavorable outcome.

Predictor	Univariate analysis			Multivariate analysis		
	OR	95% CI	*P*	OR	95% CI	*P*
**Unfavorable outcome**						
**TTR**	0,94	0.9–0.97	0.0001	0.96	0.9–0.99	<0.05
**Age**	1.06	1.02–1.09	0.01	1.05	0.99–1.11	0.09
**Female sex**	0.66	0.66–1.62	0.3			
**Admission NIHSS score**	1.43	1.22–1.67	<0.0001	1.41	1.14–1.75	0.001
**Atrial fibrillation**	2.09	1.3–3.26	0.01	1.24	0.17–6.87	0.8
**Hypertension**	2.13	0.75–6.04	0.1			
**Diabetes**	1.96	0.72–5.31	0.2			
**History of stroke**	1.06	0.41–2.73	0.8			
**Coronary heart disease**	1.25	0.51–3.01	0.6			
**Current cigarette smoking**	0.48	0.16–1.45	0.2			
**Stroke-associated infection**	1.4	0.6–4.99	0.5			
**Cardio-embolism**	2.4	0.92–6.24	0.06			
**Large-vessel occlusive etiology**	1.92	0.73–5.06	0.2			
**Small-vessel occlusive etiology**	0.24	0.06–0.97	<0.05	1.37	0.19–6.54	0.7
**Glucose**	1.15	0.9–1.46	0.2			
**Cholesterol**	0.98	0.97–1.01	0.1			
**HDL cholesterol**	0.96	0.94–1.0	<0.05	0.99	0.95–1.03	0.5
**LDL cholesterol**	0.99	0.98–1.01	0.7			
**Triglyceride**	0.99	0.98–1.0	0.02	0.99	0.97–1.0	0.4
**White cell count**	1.03	1.0–1.07	0.01	1.28	0.91–1.78	0.1
**Fibrinogen**	1.0	0.99–1.0	0.6			
**CRP**	1.24	1.07–1.45	0.001	1.16	1.03–1.51	<0.05
**fT3**	0.67	0.44–0.98	<0.05	0.64	0.42–0.99	<0.05

CI: confidence interval; CRP; C-reactive protein; fT3: free triiodothyronine; HDL: high density lipoprotein; LDL: low density lipoprotein; NIHSS: National Institute of Health Stroke Scale; OR: odds ratio; TTR: transthyretin.

In the 70 survived patients, serum TTR levels were significantly higher compared with those in 11 non-survival patients [47.4 (IQR, 37.22–61.45) mg/dl vs. 32.7 (IQR, 17.77–42.97) mg/dl (*P* = 0.009) (Fig 1B).

**Fig 2:**
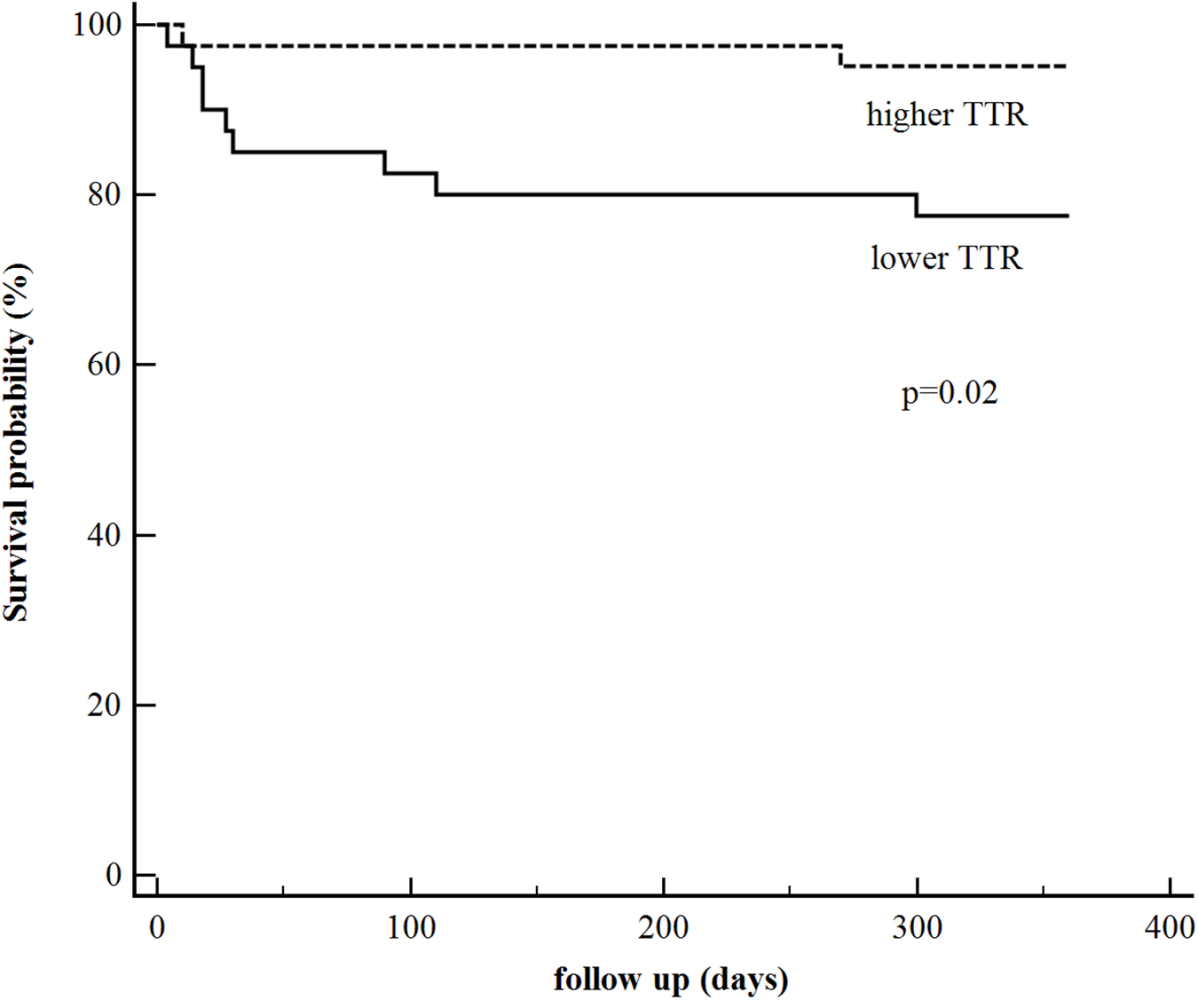
Kaplan-Meier curves of mortality in groups with higher and lower TTR (transthyretin) level (above and under the mean value– 46.9 mg/dl) (P = 0.02).

The time to death was analyzed by Kaplan–Meier survival curves based on the mean value of transthyretin levels equal to 46.9 mg/dl. Patients with lower TTR concentration had a higher risk for death, in contrast with patients in whom TTR levels exceeded 46.9 mg/dl. There were 9 deaths in the low TTR group and 2 deaths in the high TTR group (Kaplan-Meier estimations of one-year mortality 22.5% and 4.88%, respectively, hazard ratio 5.02, 95% confidence interval [1.53–16.41], *P* = 0.02) (Fig 2).

## Discussion

To our best knowledge, the current study, for the first time, investigated the interrelations between serum TTR level and one-year survival after ischemic stroke. Transthyretin was independently associated with functional outcome at discharge and one-year mortality in stroke patients, after adjusting for potential confounders. As a matter of fact, one research group explored the similar field but this study has several concerns [15]. Participants were young (the mean age was 39 years) and have been enrolled only from Chinese population. Thus the study results could be influenced by—relatively frequent in young age—cardio-embolic etiology, Moya-Moya disease (rare in other races) and some other ethnic differences (e.g. diet). Additionally, the mRS score at discharge was the primary and the only endpoint, there was not any long-term outcome, as overall one-year mortality evaluated in our study. Several possible pathophysiologic mechanisms underlying the association between low TTR level and unfavorable stroke outcome could be considered. Transthyretin is an indicator of nutritional status especially in the context of undernutrition assessment [16]. Several studies have showed that the lower serum TTR concentration is independently associated with higher mortality in severe clinical conditions as heart failure or both hemodialysis and peritoneal dialysis [17–19]. Moreover, there is a strong evidence that the malnutrition is associated with poor ischemic stroke outcome [20,21]. Therefore, the link–low TTR level, poor nutritional status, unfavorable stroke outcome, is quite conceivable.

Recent study has shown that changes in TTR concentration could be related to infections developed in the course of stroke [22]. In our group of patients, we have not observed such association. On the other hand, systemic markers of inflammation, like CRP levels and WBC count have been shown to be predictors of stroke outcome [23–25]. It is not surprising that the same observation has been noticed in our study. Transthyretin belongs to the negative acute-phase reactants meaning that following inflammation the TTR gene is downregulated and protein levels in blood become reduced [26]. The decrease of TTR levels associated with more severe stroke outcome detected in our research can be other component of acute phase response elicited in the course of critical illnesses as infection, trauma, surgery, myocardial infarction as well as ischemic stroke [27,28]. However, such effects are less probable, because TTR has very short half–life time of about 2 days [6]. Thus its relation to long-term effects can be associated with other, more persistent pathomechanism.

An interesting hypothesis which could explain the association of TTR concentration and stroke severity is based on the role of this protein in the thyroid hormones transport. Transthyretin is a carrier of these hormones from the blood via the blood–CSF barrier into the CSF [9,29]. From the experimental research we know that reduction of TTR is harmful for cell survival after an ischemic insult of the brain [30]. Other studies have proved that brain ischemia in rats caused reduction in choroid plexus blood flow and disruption of the blood-CSF barrier. This influenced on the movement of various compounds from blood to regions closed to the ventricular system and vice versa [31]. Thus damaged choroid plexus with impaired blood-CSF barrier in the course of brain ischemia might impact on thyroid hormone distribution in the CSF (and finally in brain tissue) with TTR. Such an explanation can be only hypothetical, because we have not evaluated TTR levels in CSF due to clinical contraindications–lumbar puncture is not a routine procedure in acute ischemic stroke, can be harmful particularly in patients with brain oedema or treated with oral anticoagulants.

There are few other limitations of our study. It was conducted in one hospital, the TTR levels were measured only once, and we cannot identify cause and effect relationship. The patients sample was relatively small because of strict inclusion and exclusion criteria, which may additionally limit the ability to generalize the results of this report. However, exploration conducted on homogeneous population allow to avoid possible confounding factors.

In conclusion, our study suggests that the serum TTR level status is associated with the functional outcome in patients with acute ischemic stroke. These findings may be useful for risk stratification of ischemic stroke patients. More extensive investigations are warranted, especially with the repetitive assessment of TTR concentration not only in the blood but even preferably in CSF.
